# Voice quality after transoral CO_2_ laser microsurgery (TOLMS): systematic review of literature

**DOI:** 10.1007/s00405-022-07418-3

**Published:** 2022-05-03

**Authors:** Andrea Colizza, Massimo Ralli, Chiara D’Elia, Antonio Greco, Marco de Vincentiis

**Affiliations:** 1 Department of Sense Organs, Sapienza University of Rome, Viale del Policlinico 155, 00186 Rome, Italy; 2 Otorhinolaryngology Unit, Policlinico Umberto I, Rome, Italy; 3 Department of Oral and Maxillofacial Sciences, Sapienza University of Rome, Rome, Italy

**Keywords:** Laser microsurgery, Laryngeal cancer, Voice outcomes, Systematic review

## Abstract

**Purpose:**

Transoral laser microsurgery (TOLMS) with carbon dioxide is a safe approach for laryngeal carcinoma. In literature there are three main methods for evaluating speech outcomes: acoustic and aerodynamics analysis, perceptual evaluation and patient-reported outcomes (PROs). The aim of this study was to systematically review the literature about the voice quality outcomes of TOLMS according to type of cordectomy.

**Methods:**

A systematic literature review was performed and all the results until December 2021 were extrapolated. We evaluated the acoustic and aerodynamics parameters (fundamental frequency, harmonics to noise ratio, jitter, shimmer and maximum phonation time), perceptual data (GRBAS scale) and patient-related outcomes (VHI scale).

**Results:**

24 studies met the inclusion criteria for a total number of 1207 patients enrolled. The number for each type of cordectomy are: 287 type I (23.78%), 311 type II (25.78%), 328 type III (27.14%), 129 type 4 (10.69%) and 152 type V (12.60%). Patients are grouped according to the type of cordectomy in: limited cordectomy (type I and II) and extended cordectomy (types III–IV–V). The difference between two groups is statistically significative in terms of acoustic analysis, perceptual data and patient-related outcomes (*p* < 0.05).

**Conclusions:**

Patients who underwent type I or II cordectomy have significantly better quality of voice in terms of VHI, perceptual voice quality evaluations and acoustic parameters compared to type III, IV and V cordectomies. The effect of TOLMS on the voice should depend from the extent of the resection and in particular from the scar of the vocal muscle.

## Introduction

Transoral laser microsurgery (TOLMS) with carbon dioxide (CO_2_) is a safe approach for laryngeal squamous cell carcinoma (LSCC) [1, 2] and is considered, such as the radiotherapy treatment, the standard of care for primary early stage (T1 and T2) LSCC. Several types of TOLMS are described and are classified according to the European Laryngological Society (ELS) Classification [3]. The laser CO_2_ cordectomy are: Type I subepithelial, Type II subligamental, Type III transmuscolar, Type IV total and Type V extended.

In literature the oncological outcomes of TOLMS have been extensively investigated by systematic reviews and meta-analysis. In fact, is clear that TOLMS and radiotherapy for early stage of LSCC are similar for overall survival [4], but TOLMS is favored due to lower costs and fewer post-treatment adverse events [5].

About the functional and vocal outcomes after TOLMS, in literature are present a lot of case series papers about single center experience, but at the best of our knowledge, is not present a systematic review on this topic. In literature there are three main methods for evaluating speech outcomes: acoustic analysis, perceptual evaluation [8] and patient-reported outcomes (PROs) [9]. The goal of the study is to review the literature regarding laser cordectomy to outline the voice profile in the various degrees of this type of intervention.

## Materials and methods

### Literature search strategy

Following the Preferred Reporting Items for Systematic and Meta-Analyses (PRISMA) guidelines, a systematic literature review was performed using MEDLINE, EMBASE, PubMed and Scopus databases. The search strategy was conducted using combinations of the following terms: “cordectomy and acoustic analysis” OR “cordectomy GRBAS” OR “cordectomy VHI” OR “cordectomy voice outcome” OR “vocal laser surgery GRBAS” OR “vocal laser surgery VHI”.

### Study selection

For this review we considered and extrapolated all the results until December 2021. The papers considered for this review reported: abstract available in English language, type of cordectomy according to ELS and the results of voice outcomes after 6 months to 1 year from TOLMS (acoustic analysis, perceptual evaluation and PROs). We enrolled studies also with some of these analyses, because in literature, in general, there are present papers that analyzed just a few of speech outcomes.

We excluded articles with lacking information regarding the type of cordectomy, time of voice evaluation, previous RT treatment on the larynx and article in other languages or abstract unavailable.

Title and abstract were watchfully examined by two authors (A.C and C.D.E) independently, and disagreements were resolved by a discussion with a third author (M.R).

### Data extraction

The full text of the included studies was reviewed and data extraction was performed using a standard registry database. The data registered in each case were: number of patients, type of cordectomy, acoustic and aerodynamics analysis (fundamental frequency, harmonics to noise ratio, jitter, shimmer and maximum phonation time), perceptual data (GRBAS scale) and PROs (VHI scale).

### Voice outcomes

In this review we considered some outcomes widely used in literature. In the following section we introduce them and in Table 1 are resumed these parameters.

**Table 1:** Parameters of voice outcomes analysed in this study

Acoustic Analysis	Fundamental Frequency (F0): result of the rate of vibration of the (neo) glottis which oscillate in the airflow when appropriately tensed
	Harmonics to Noise Ratio (HNR): ratio between the total energy of the periodic voice signal and the energy of noise components
	Jitter: relative variability in the F0 between contiguous (neo) glottal cycles
	Shimmer: relative variability in the amplitude of sound waves
Aerodynamics Parameter	Maximum Phonation Time (MPT): the longest period during which a patient can sustain phonation of a vowel sound, typically /a/
Perceptual Evaluation	GRBAS: Grade, Roughness, Breathiness, Asthenia, Strain scale assessment
Patient-Reported Outcomes (PROs)	VHI: Voice Handicap Index

### Acoustic and aerodynamics analysis

Quantitative acoustic measurements are more regularly studied and are obtained from tools that digitize and analyze the voice and quantify the characteristics [6, 7]**.**

The parameters considered in this review are: fundamental frequency (F0), Noise Harmonics to Ratio (NHR), Jitter% (Jitt), Shimmer% (Shim).

The aerodynamics parameter is the maximum phonation time (MPT) measured (in seconds) with the pronunciation of/aa/as the primary value.

These outcomes are indicated in literature to obtain information about the pitch, the stability and the amount of noise components [8, 9].

### Perceptual analysis

Another of the factors to take into account is the auditive perception generated in the listener and is evaluated by ways of perceived voice quality.

For the perceptual outcomes we have considered a well-established assessment tool, such as the GRBAS [10] scale in which are estimates the grade of hoarseness (G), roughness (R), breathiness (B), asthenia (A) and strain in the voice (S) on a scale from 0 to 3 (0, normal; 1, mild; 2, moderate; 3, severe).

### Patient-reported outcomes (PROs)

The patient’s perceived voice quality in literature is evaluated questionnaires that give an idea of the subjective impact that a vocal problem produces in the subject. The patient-reported outcomes (PROs) mostly used in literature is the VHI scale [11].

The VHI consists in a questionnaire of 30 items: 10 items on emotional issues, 10 items on physical issues, and 10 items on functional issues. Scoring is from 0 to 120, with 120 representing the maximum perceived disability. Each item is scored on a four point scale: 0 = never, 1 = almost never, 2 = sometimes, 3 = almost always, and 4 = always. A VHI score of 0–40 points indicate a handicap of slight impact, a score of 41–60 indicates moderate impact, and a score of 61 indicates severe impact.

### Statistical methods

All statistical analyses were performed using SPSS Version 25.0 (IBM Corp, Armonk, NY, USA). Descriptive analyses were mainly applied. Data are indicated as mean, range and percentage. Student *t* test was used for outcome comparisons between groups of patients. A level of significance of *p* < 0.05 was used.

## Results

### Search results, data synthesis and analysis

**Fig. 1:**
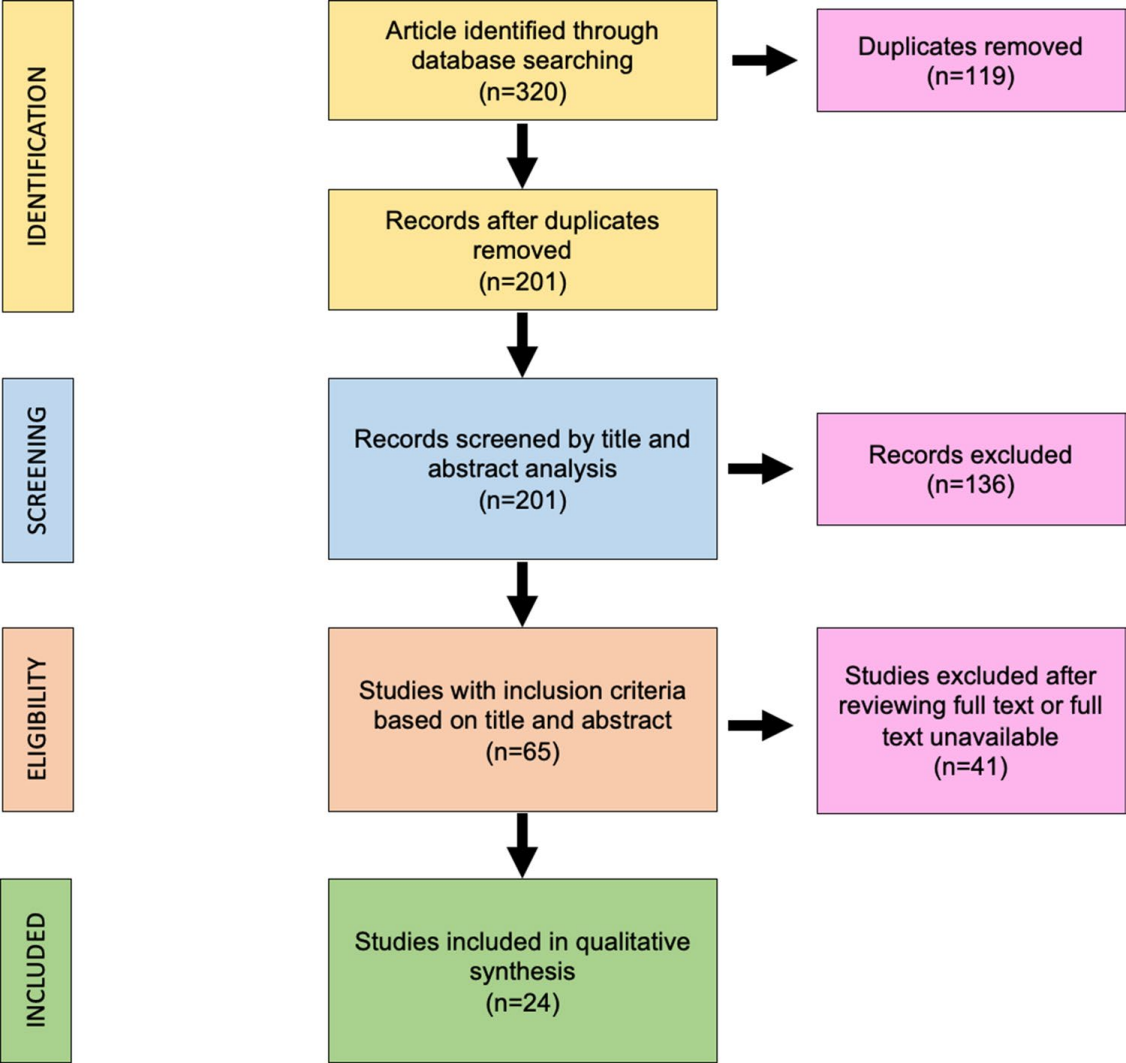
Preferred Reporting Items for Systematic Reviews and Meta-Analyses (PRISMA) diagram followed in this review. The diagram shows the information flow through the different phases of the review and illustrates the number of records that were identified and included

The search algorithm and review results are outlined in Fig. 1.

**Fig. 2:**
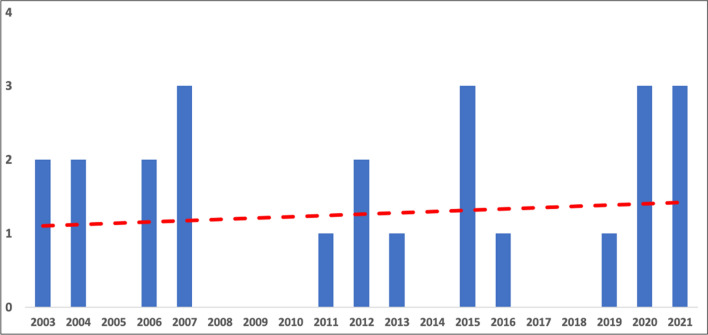
Histogram with the number of papers for every year. In red is the trend line of published articles considered in this review

**Fig. 3:**
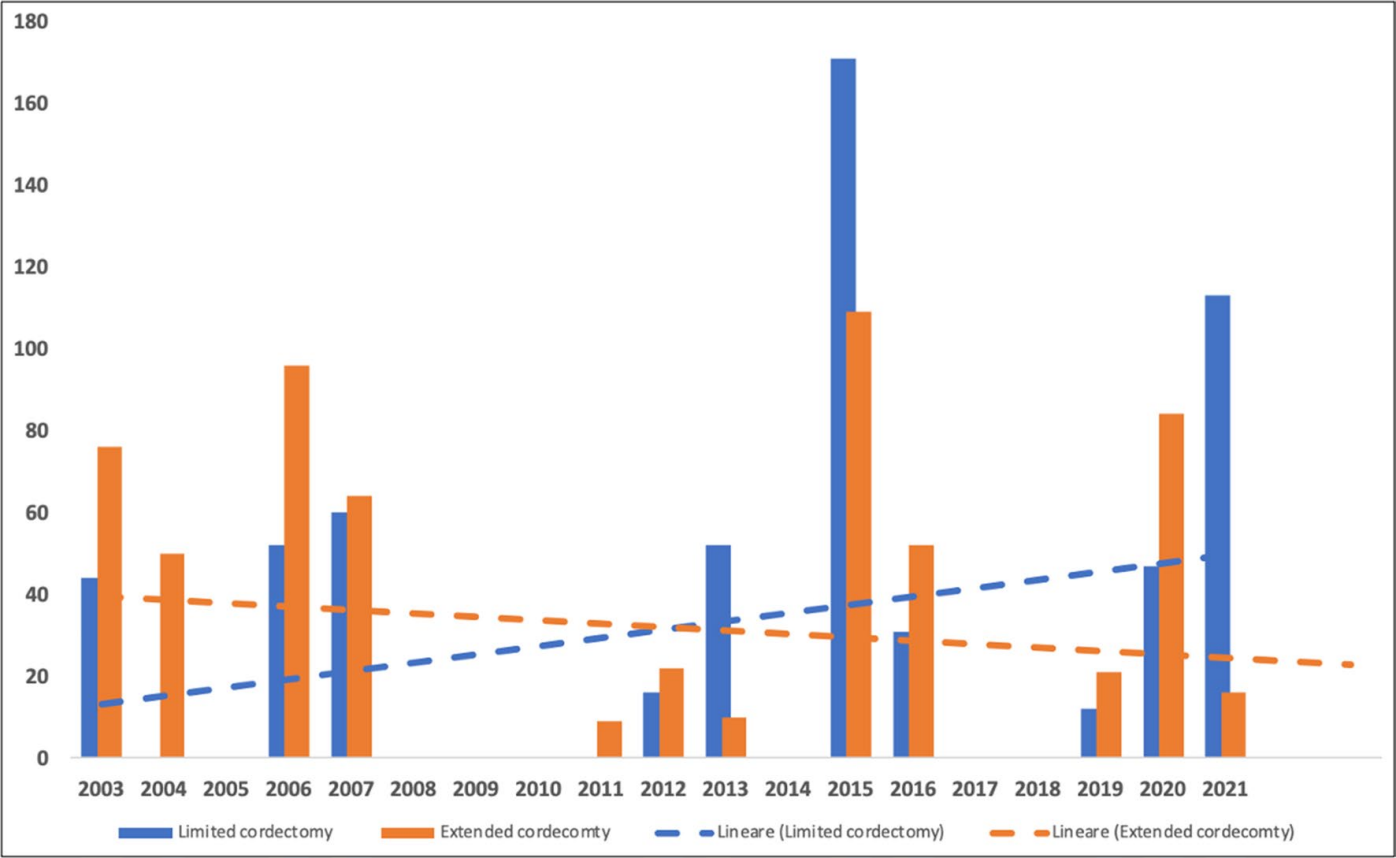
Histogram of type of cordectomy (limited or extended) for every year. The trend lines show an increment of limited cordectomy and a progressive reduction of extended cordectomy

The initial search found 320 studies on the MEDLINE database, EMBASE, Scopus and the Cochrane Library databases. The removal of duplicates identified 201 publications. All the 201 papers were screened in title and abstract, and 65 manuscripts were reviewed in full text. Of these, 24 studies met the inclusion criteria, while the remaining 41 studies were excluded. The included studies were published in peer-reviewed journals. The data collected from each study were transcribed in a tabular form. In Fig. 2 is report the histogram with the number of studies considered for years of publication, and in Fig. 3, the type of cordectomy performed along the time.

Studies considered in this review, the total number of patients for each study and the number of patients according to the type of cordectomy are reported in Table 2.

**Table 2:** Papers considered in this review and patients subdivided according to ELS cordectomy classification

Author	Year	Total patients of the study	Type of cordectomy according to ELS classification	Number of patients
Peretti et al. [12]	2003	69	Type I	7
			Type II	11
			Type III	21
			Type IV	14
			Type V	16
Peretti et al. [13]	2003	51	Types I–II	26
			Types III–IV–V	25
Krengli et al. [14]	2004	30	Types III–IV	30
Policarpo et al. [15]	2004	20	Type III	7
			Type IV	13
Haddad et al. [16]	2006	15	Type I	3
			Type II	5
			Type III	4
			Type IV	3
Ledda et al. [17]	2006	133	Type I	16
			Type II	28
			Type III	31
			Type IV	13
			Type V	45
Roh et al. [18]	2007	75	Types I–II	45
			Types III–IV	17
			Type V	13
Xu et al. [19]	2007	30	Types III–IV	30
Vilaseca et al. [20]	2007	19	Types I–II	15
			Type V	4
Chu et al. [21]	2011	9	Type III	9
Chu et al. [22]	2012	25	Types I–II	13
			Types III–IV–V	12
Galletti et al. [23]	2012	13	Type II	3
			Type III	6
			Type IV	4
Bahannan et al. [24]	2013	62	Types I–II	52
			Types III–IV–V	10
Bertino et al. [25]	2015	101	Types I–II	66
			Types III–IV	35
Greulich et al. [26]	2015	179	Types I–II	105
			Type III	74
Lee et al. [27]	2016	57	Types I–II	21
			Types III–IV–V	36
Fink et al. [28]	2016	26	Type I	7
			Type II	3
			Type III	12
			Type V	4
Del Mundo et al. [29]	2019	33	Type I	8
			Type II	4
			Type III	21
Hamzany et al. [30]	2020	55	Types I–II	34
			Types III–IV–V	21
Kosztyła-Hojna et al. [31]	2020	30	Type III	13
			Type IV	6
			Type V	11
Şencan et al. [32]	2020	46	Types I–II	13
			Type III	16
			Types IV–V	17
Lechien et al. [5]	2021	60	Type I	30
			Type II	30
Song et al. [33]	2021	51	Type I	24
			Type II	18
			Type III	9
Staníková et al. (34)	2021	18	Types I–II	11
			Types III–IV–V	7

### Study cohort

A total of 1207 patients were enrolled in this review. The number of patients for each type of cordectomy are: 287 type I (23.78%), 311 type II (25.78%), 328 type III (27.14%), 129 type 4 (10.69%) and 152 type V (12.60%).

In almost all studies considered for the vocal analysis the patients are grouped according to the type of cordectomy in: limited cordectomy (type I and II) and extended cordectomy (types III–IV–V). For this reason, in this we review we follow the same division to create a population of patients as homogeneous as possible. Among the limited cordectomy we found 598 (49.5%) patients and for extended cordectomy 609 (50.5%). The study cohort characteristics are resumed in Table 3.

**Table 3:** Study cohort characteristics

Total patients	1207
Type of cordectomy	
• Type 1	287 (23.78%)
• Type 2	311 (25.78%)
• Type 3	328 (27.14%)
• Type 4	129 (10.69%)
• Type 5	152 (12.6%)
Limited cordectomy	
• Type I + II	598 (49,5%)
Extended cordectomy	
● Type III + IV + V	609 (50,5%)

### Voice outcomes

Subjective and objective voice quality outcomes after limited or extended cordectomy are resumed in Table 4. The difference of every parameter is statistically significative between two groups; the only parameter that is not statistically significative is the Harmonics to Noise Ratio (HNR). About the acoustic outcomes the mean F0 for limited cordectomy is 153.57 ± 18.01 Hz and for extended cordectomy is 171.13 ± 22.3 Hz. The difference was statistically significative (*p* < 0.05). The difference for jitter, shimmer and MPT is statistically significative between the two groups of cordectomy.

**Table 4:** Vocal outcomes in the sub-group of cordectomy

	Limited cordectomy	Extended cordectomy	*p* (95% CI)
Acoustic parameters			
• F0 (Hz)	153.57 ± 18.01	171.13 ± 22.32	*p* = 0.02^*^
• HNR • Jitter (%)	0.22 ± 0.20 1.61 ± 0.72	0.36 ± 0.31 3.43 ± 2.33	*p* = 0.193^**^ *p* = 0.0013^*^
• Shimmer (%)	6.66 ± 4.13	10.77 ± 4.88	*p* = 0.0046^*^
Aerodynamics parameter			
• MPT (seconds)	13.87 ± 2.73	9.68 ± 3.22	*p* = 0.002^*^
GRBAS scale			
• Grade of hoarseness (G)	1.16 ± 0.34	1.65 ± 0.37	*p* = 0.0008^*^
• Roughness (R)	1.08 ± 0.23	1.64 ± 0.76	*p* = 0.037^*^
• Breathiness (B)	0.78 ± 0.41	1.33 ± 0.61	*p* = 0.0051^*^
• Asthenia (A)	0.35 ± 0.39	0.88 ± 0.63	*p* = 0.0233^*^
• Strain in the voice (S)	0.70 ± 0.43	1.34 ± 0.92	*p* = 0.0277^*^
• Total	4.09 ± 0.79	6.25 ± 2	*p* = 0.0009^*^
VHI scale	15.09 ± 6.77	28.67 ± 12.46	*p* = 0.0012^*^

^*^p statistically significative

^**^p no statistically significative

The GRBAS scale total is statistically different (*p* = 0.0009) and similarly each parameter is statistically different. The mean value of VHI evaluation for limited cordectomy is 15.09 ± 6.77 and for extended cordectomy is 28.67 ± 12.46 and the difference is statistically significative (*p* = 0.0012).

## Discussion

The best objective to achieve during microsurgery on larynx is a compromise between oncological radicality, vocal fold function and impact on the quality of life of the patients [35]. In general, in the field of oncological surgery, quality of voice is secondary to the radical oncological excision of the neoplasm. However, is important to analyze the impact on the vocal outcome and quality of life.

Almost all papers on this topic present in literature, comparing the voice outcomes after TOLMS and RT in LSCC treatment. The results are contradictory; some works show a better voice outcome after TOLMS [36, 37], some better voice after RT [38, 39] and other found no statistically significant difference between RT and TOLMS [40].

To date in literature is not present a systematic review analyzing vocal outcomes according to each type of cordectomy. In this review we analyze only the most frequent parameters reported in literature, but one of the most important and useful tools in the assessment of the voice function is laryngophotography. This includes: High definition laryngophotography, Ultra high speed laryngophotography to study the various parameters of the glottic wave which relates well to the quality of voice, as well as Laryngostroboscopy [41].

When comparing the voice parameters of limited cordectomy (subepithelial and subligamental) and extended cordectomy (transmuscolar, total and extended), the results are better for limited: F0, Jitter and Shimmer are statistically different from extended cordectomy. The only acoustic parameter that is not better is the HNR. The Aerodynamics parameter (MPT) is statistically different between the two group of cordectomies too.

Voice one of the most important relational tools and the auditive perception generated in the listener is an outcome analyzed in literature. For this reason, some scales are proposed and approved, such as GRBAS [10] and IINFV0 [42, 43]. The most diffuse in literature is GRBAS and in this review we found a statistically significative difference between limited and extended cordectomy for each parameter (G, R, B, A, S) and for the total score.

The last parameters are the patient-reported outcomes (PROs) and represent the patient’s perceived voice quality. In this review we considered the Voice Handicap Index, the scale largely diffuses in literature. Despite the spread of rapid and economic self-evaluation methods, such as VHI, is important to underline that the questionnaires collecting data about the patient's own evaluation of voice is very subjective, and liable to uncertainties and fallacies.

The VHI scale analysis shown, such as GRBAS and acoustic parameters, a statistically significative difference from limited to extended cordectomy.

These results in each field (acoustic parameters, perceptual analysis and PRO’s) between the extension of cordectomy should be explained by the alteration and subsequent regeneration of laryngeal structures, such as vocal ligament and vocal muscle (thyro-arytenoid muscle).

The cordectomies I and II reach the tissues superficial to the vocal ligament. Any deeper excision shall traumatize the vocal ligament leading to healing by fibrosis which will affect the free mobility of the vocal fold mucosa on the deeper structures, leading to various degrees of derangement of the vocal function. Limited cordectomies shall derange the voice function due to interference with the layered structure of the vocal fold, creating scars of various extends and depths. These scars most probably disturb the regular glottic wave, thus affecting the voice quality. On the other hand, extended cordectomies, shall add an important organic factor to the derangement in addition to the disturbed vocal fold layered structure and scarring. This added factor is the reduction of the bulk of the vocal fold due to the excision of parts of the thyro-arytenoid muscle, leaving the glottis with one vibrator, the healthy non-operated vocal fold.

The vocal muscle does not participate in the vibratory mechanism of voice production, but it helps in the approximation of the vocal folds leading to good closure of the glottis allowing the aerodynamic processes to produce good vibrations at the vocal folds' mucosal edges. The scarring of the vocal muscle may not be the mechanism worsening of the voice results with the extended cordectomy group.

The future prospects are approaches of regenerative medicine to improve the vocal fold vibration and muscle contraction.

## Conclusions

This review underlines that patients who underwent type I or II cordectomy have significantly better quality of voice in terms of VHI, perceptual voice quality evaluations and acoustic parameters compared to type III, IV and V cordectomies. The effect of TOLMS on the voice should depend from the extent of the resection and in particular from the scar of the vocal muscle. At the best of our knowledge this is the first review comparing the vocal outcomes after cordectomies according to the type of resection.
